# Supplementary material for: Longitudinal Trajectories of Alcohol Consumption with All-Cause Mortality, Hypertension, and Blood Pressure Change: Results from CHNS Cohort, 1993–2015
Source: Nutrients. 2022 Nov 29;14(23):5073. doi: 10.3390/nu14235073 (PMC9739068; doi:10.3390/nu14235073)
Supplement: Supplementary file 1 [file nutrients-14-05073-s001.zip › nutrients-2035198-supplementary.pdf]

Supplemental Materials

Methods

Statistical analysis

To detect distinct trajectories of longitudinal alcohol consumption development, group-based trajectory modeling (GBTM) was performed with the ‘traj’ command[1] in STATA to identify subgroups of CNHS participants who shared similar underlying trajectories between 1993 and 2015. GBTM is a specialized form of finite mixture modeling used to identify groups of individuals who share similar developmental trajectories over time[2]. It uses maximum likelihood to estimate model parameters and handle incomplete data without listwise deletion. To fulfill the requirement of the censored normal distribution model in GBTM, participants who reported not drinking in each survey were categorized as non-drinker and excluded from the model, and the rest of individuals were then included in GBTM. Each model accuracy was based on standard criteria[2, 3], including the Bayesian information criterion (BIC), which accounts for the goodness-of-fit of the models, and the posterior probability, which accounts for internal reliability. We compared the BIC values to the previous model and 2*ΔBIC over 10 was regarded as better goodness-of-fit indicating significant change. Analysis of trajectory calculated the posterior probability for each participant (from 0 to 1) and assign them to the group with the highest probability. We also maintained the average of posterior probability of assignments above 0.7 in all cases and group sizes above 5% of participants, which are regarded as acceptable[2]. Finally, we used a graphical presentation approach to plot distinct trajectories in the final model for each class with time, and assigned labels to trajectories based on their graphic patterns.

In trajectory analysis, participants with at least three alcohol intake records and at least one being from 1993 were included in the current study for reliability. In the first step in the GBTM, the number of trajectories was chosen using a quadratic (x^2) model (Table S1 in the Supplemental Material). As stated above, the selection of the number of trajectory groups were based on goodness-of-fit (2*ΔBIC) (Table S1), average posterior probability (above 0.7) (Table S2 and Table S3), group size (above 5% of all population) and clinical plausibility. The shape of each trajectory group was then decided by testing linear, quadratic, cubic, quartic, and quintic terms of time and retaining the highest term when significant.

A total of 3060 participants with at least three measurements of alcohol consumption included in the GBTM analysis (Figure S1). In trajectory modeling, better goodness-of-fit (2*ΔBIC) remained significant with the number of trajectory group increments (Table S1). At the same time, maintaining an average posterior probability of >0.7 and each group size of >5% (Table S2) was considered acceptable in every solution. Three-group or fewer trajectory solutions met all the above requirements; hence, we chose a three-trajectory solution as the optimal number for trajectory groups. We then tested the linear, quadratic, cubic, quartic, and quintic terms of time for three-trajectory solution shapes and a final three-trajectory group with the “5,2,4” model for alcohol consumption was chosen. The distinct trajectories were plotted with time (Figure 1) and were assigned respective labels (Table S3): (i) Light-drinker (N=1712, 56.0%): alcohol consumption was maintained at a light level (less than 10 units per week in the long term); (ii) Moderate-drinker (N=1169, 38.2%): alcohol consumption was maintained at a moderate level (approximately 20 units per week); (iii) Heavy-drinker (N=179, 5.9%): alcohol consumption was maintained at a high level for a long time (more than 40 units per week) and decreased but still remained the highest level at last. Finally, 5298 participants were divided into 4 classes: non-drinker, light drinker, moderate-drinker, and heavy-drinker.

Supplemental Table S1. Characteristics for Trajectory Modeling for Trajectory on Alcohol Consumption.

Total Alcohol Consumption	LOGLIK	BIC	2*ΔBIC	AIC
2 trajectory – x^2	-55218.27	-55250.38	-	-55226.27
3 trajectory – x^2	-54834.87	-54883.02	734.72	-54846.87
4 trajectory – x^2	-54625.93	-54690.14	385.76	-54641.93
5 trajectory – x^2	-54466.20	-54546.46	287.36	-54486.20
6 trajectory – x^2	-54466.20	-54562.51	32.1	-54490.20
7 trajectory – x^2	-54359.56	-54471.93	181.16	-54387.56

LOGLIK, log-likelihood; BIC, Bayesian Information Criteria; 2*ΔBIC, change of the BIC (compared to the preceding BIC) multiplied two; AIC, Akaike Information Criteria.

Supplemental Table S2. Comparison of Total Alcohol Consumption Trajectory Model Posterior Probabilities.

	N (%)	Mean (SD)	Min	Max
Posterior probabilities using **two** trajectory model (x^2-term for each trajectory)				
Group 1	2283 (74.6)	0.94 (0.11)	0.50	1.00
Group 2	777 (25.4)	0.90 (0.14)	0.51	1.00
Posterior probabilities using **three** trajectory model (x^2-term for each trajectory)				
Group 1	1686 (55.1)	0.89 (0.14)	0.50	1.00
Group 2	1182 (38.6)	0.85 (0.14)	0.50	1.00
Group 3	192 (6.3)	0.88 (0.16)	0.50	1.00
Posterior probabilities using **four** trajectory model (x^2-term for each trajectory)				
Group 1	1617 (52.8)	0.89 (0.14)	0.50	1.00
Group 2	1183 (38.7)	0.84 (0.15)	0.42	1.00
Group 3	47 (1.5)	0.89 (0.17)	0.43	1.00
Group 4	213 (7.0)	0.88 (0.16)	0.49	1.00
Posterior probabilities using **five** trajectory model (x^2-term for each trajectory)				
Group 1	1601 (52.3)	0.88 (0.14)	0.48	1.00
Group 2	1171 (38.3)	0.84 (0.15)	0.42	1.00
Group 3	46 (1.5)	0.90 (0.15)	0.51	1.00
Group 4	187 (6.1)	0.88 (0.15)	0.34	1.00
Group 5	55 (1.8)	0.85 (0.20)	0.37	1.00
Posterior probabilities using **six** trajectory model (x^2-term for each trajectory)				
Group 1	0 (0)	-	-	-
Group 2	1601 (52.3)	0.88 (0.14)	0.48	1.00
Group 3	1171 (38.3)	0.84 (0.15)	0.42	1.00

Group 4	46 (1.5)	0.90 (0.15)	0.51	1.00
Group 5	187 (6.1)	0.88 (0.15)	0.34	1.00
Group 6	55 (1.8)	0.85 (0.20)	0.37	1.00
Posterior probabilities using **seven** trajectory model (x^2-term for each trajectory)				
Group 1	4 (0.1)	0.98 (0.03)	0.95	1.00
Group 2	1539 (50.3)	0.87 (0.14)	0.48	1.00
Group 3	1173 (38.3)	0.82 (0.14)	0.36	1.00
Group 4	209 (6.8)	0.83 (0.17)	0.43	1.00
Group 5	44 (1.4)	0.89 (0.17)	0.38	1.00
Group 6	56 (1.8)	0.86 (0.18)	0.37	1.00
Group 7	35 (1.1)	0.90 (0.13)	0.55	1.00

SD, standard deviations; Min, minimum; Max, maximum

Values are means (standard deviations) for posterior probabilities, minimum and maximum posterior probabilities, and numbers (percentages) for categorical variables.

Supplementary Table S3. Posterior Probabilities for Total Alcohol Consumption Trajectories Using Three Trajectory Model ("5,2,4" model).

	**N (%)**	**Mean (SD)**	**Min**	**Max**
Group 1 (Light Drinker)	1712 (56.0)	0.89 (0.14)	0.50	1.00
Group 2 (Moderate Drinker)	1169 (38.2)	0.85 (0.15)	0.50	1.00
Group 3 (Heavy Drinker)	179 (5.9)	0.89 (0.15)	0.50	1.00

SD, standard deviations; Min, minimum; Max, maximum

Values are means (standard deviations) for posterior probabilities, minimum and maximum posterior probabilities, and numbers (percentages) for categorical variables.

Supplemental Figure S1. Study Flowchart.

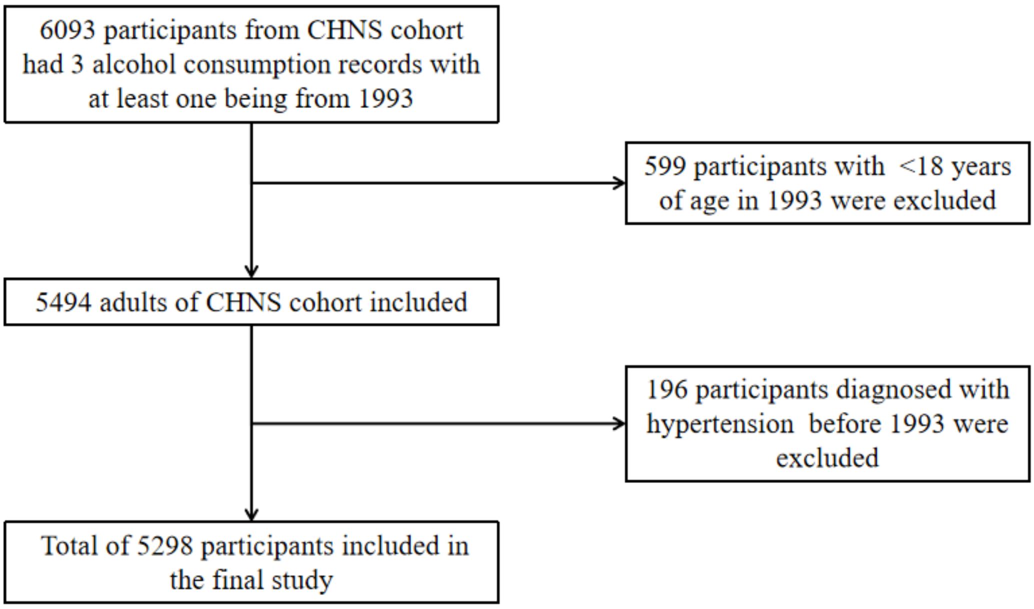

CHNS, the China Health and Nutrition Survey

Supplemental Figure S2. The Association between Alcohol Consumption and Change in Diastolic Blood Pressure.

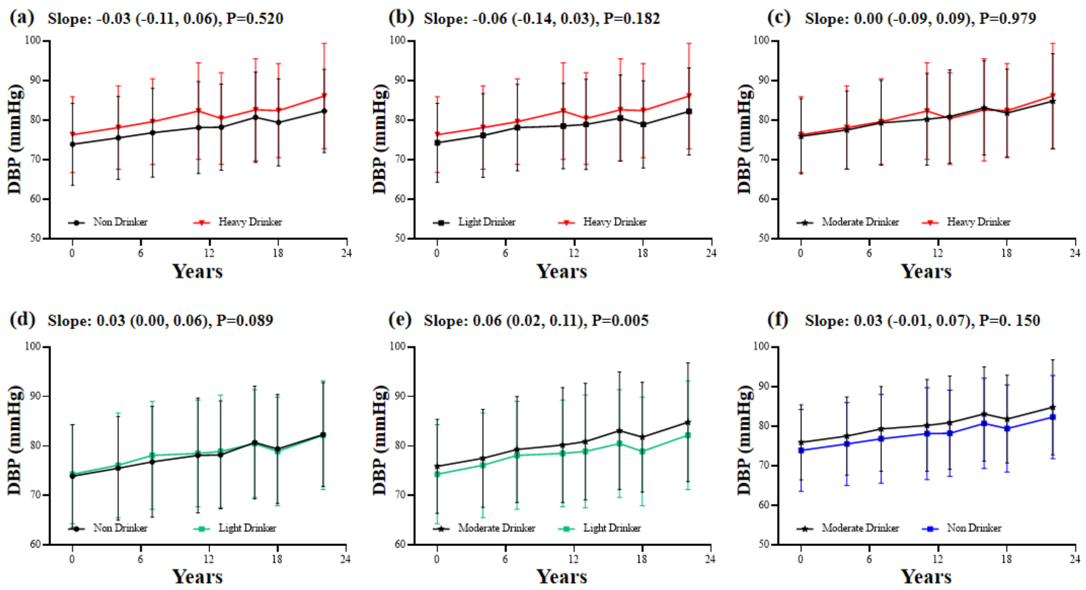

The multivariate-adjusted generalized linear mixed-effects models were adjusted for age, sex, marital status, education, residence area, smoking status, mean BMI, mean waist circumference.

P-value <0.017 was considered statistically significant for the multiple comparisons.

Supplemental Figure S3. Histogram of Residuals (A) and The Residuals Plot (B) for Systolic Blood Pressure.

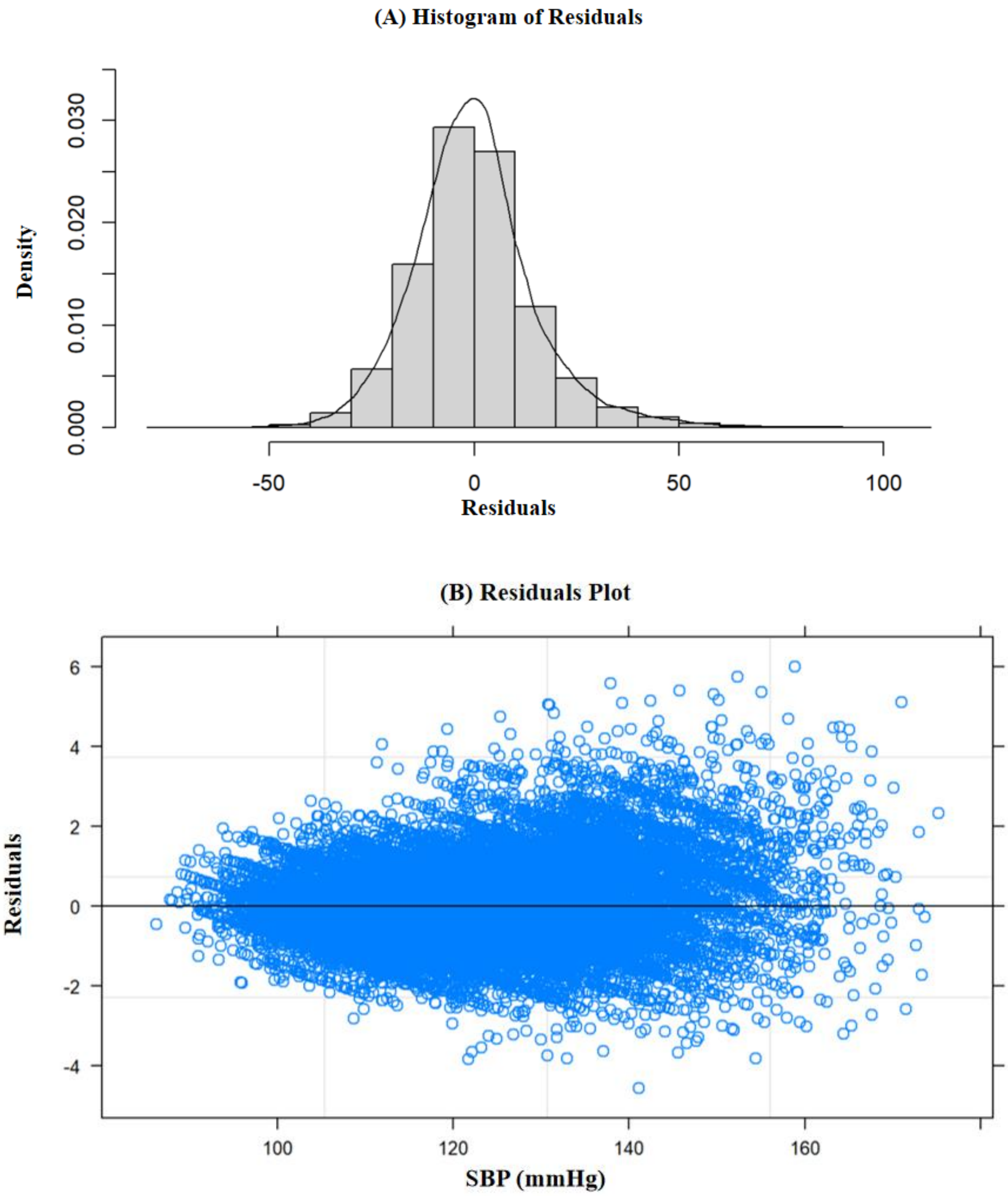

SBP, systolic blood pressure

Supplemental Figure S4. Histogram of Residuals (A) and The Residuals Plot (B) for Diastolic Blood Pressure.

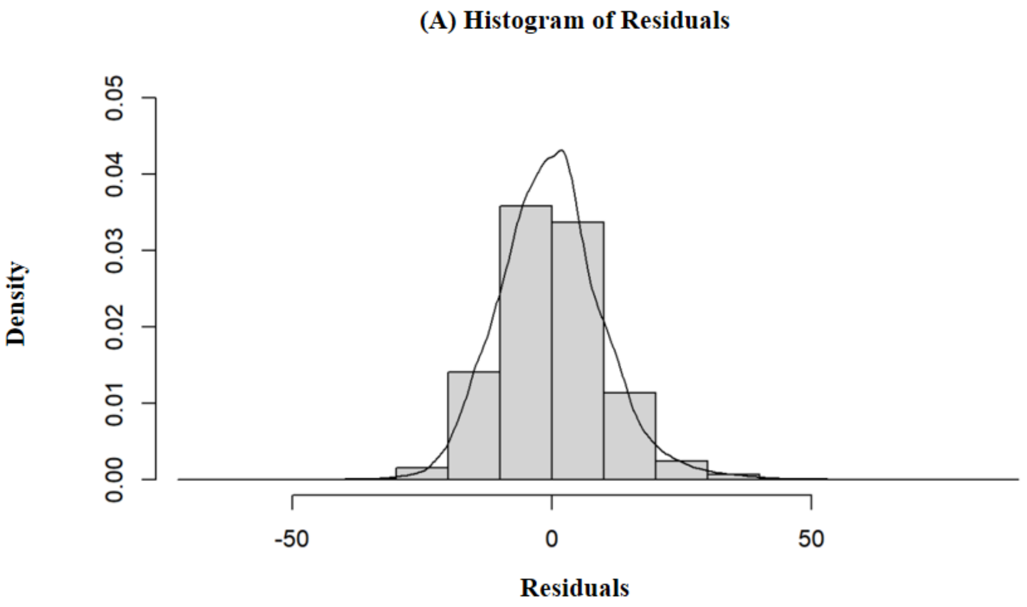

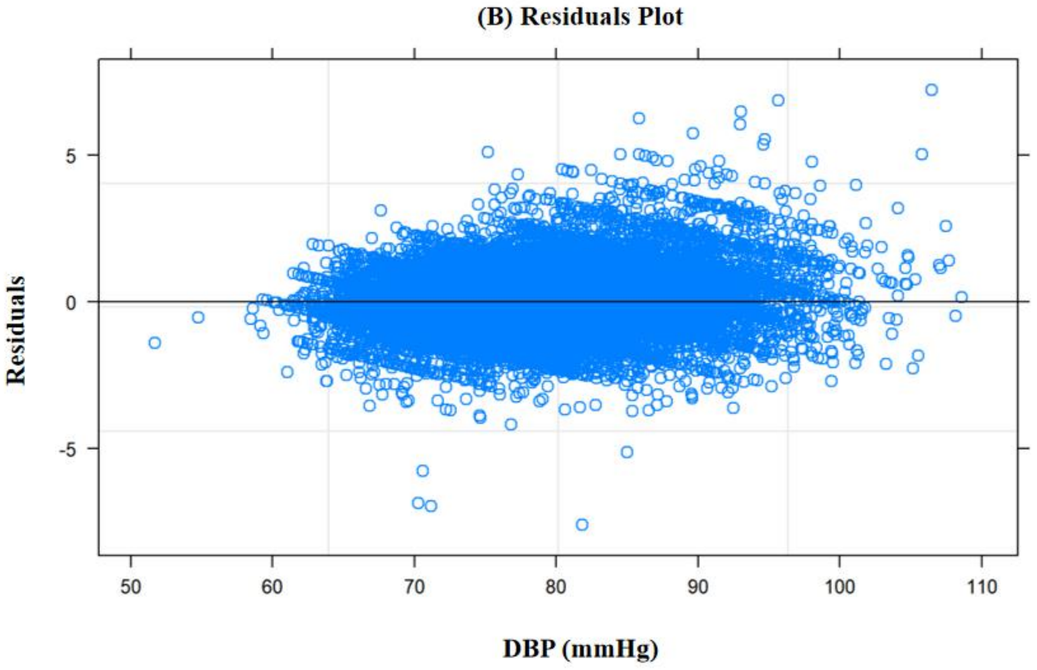

DBP, diastolic blood pressure

Supplemental Figure S5. Histogram of Residuals (A) and The Residuals Plot (B) for Pulse Pressure.

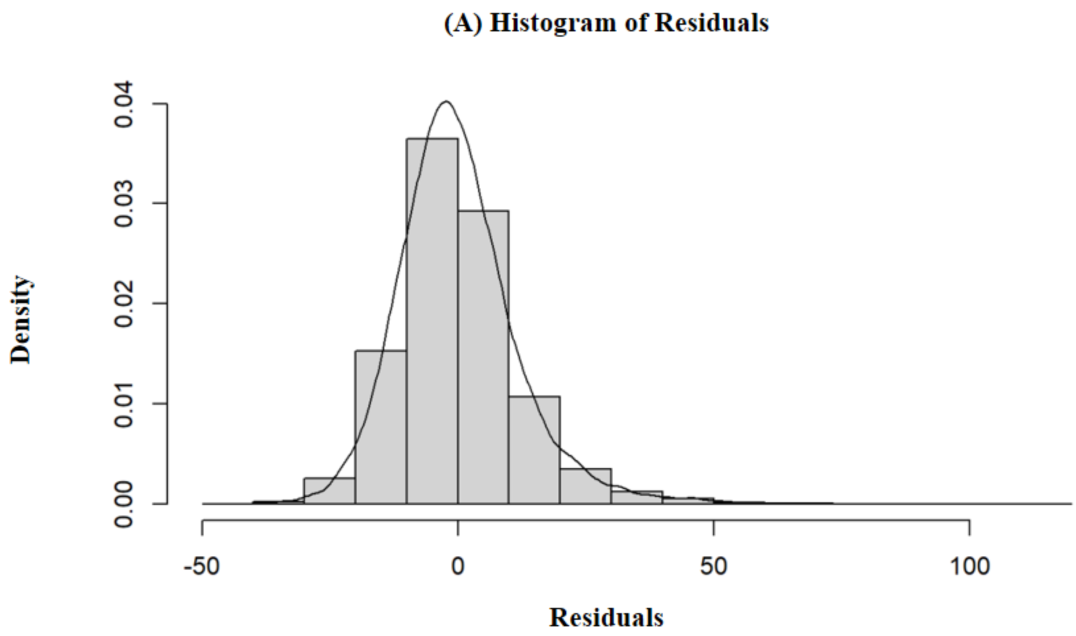

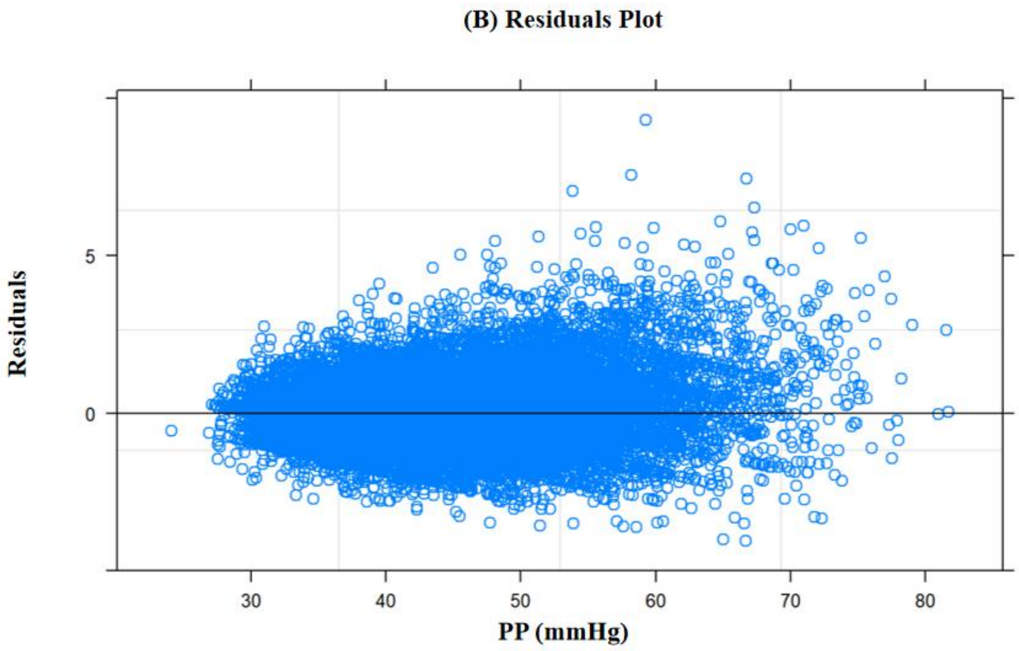

PP, pulse pressure

References:

1. Jones BL, Nagin DS. A note on a stata plugin for estimating group-based trajectory models. *Sociological Methods & Research*. 2013;42:608-613
2. Anderson-Cook, Christine, M. Group-based modeling of development. *Journal of the American Statistical Association*. 2006
3. Nagin D, Odgers C. Group-based trajectory modeling in clinical research. *Annual review of clinical psychology*. 2010;6:109-138
